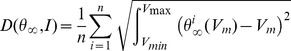# Spike-Threshold Adaptation Predicted by Membrane Potential Dynamics *In Vivo*

**DOI:** 10.1371/journal.pcbi.1003560

**Published:** 2014-04-10

**Authors:** Bertrand Fontaine, José Luis Peña, Romain Brette

**Affiliations:** 1 Dominick P. Purpura Department of Neuroscience, Albert Einstein College of Medicine, Bronx, New York, United States of America; 2 Laboratoire Psychologie de la Perception, CNRS and Université Paris Descartes, Paris, France; 3 Département d'Etudes Cognitives, Ecole Normale Supérieure, Paris, France; 4 Sorbonne Universités, UPMC Univ. Paris 06, UMR_S 968, Institut de la Vision, Paris, France; 5 INSERM, U968, Paris, France; 6 CNRS, UMR_7210, Paris, France; Duke University, United States of America

## Abstract

Neurons encode information in sequences of spikes, which are triggered when their membrane potential crosses a threshold. *In vivo*, the spiking threshold displays large variability suggesting that threshold dynamics have a profound influence on how the combined input of a neuron is encoded in the spiking. Threshold variability could be explained by adaptation to the membrane potential. However, it could also be the case that most threshold variability reflects noise and processes other than threshold adaptation. Here, we investigated threshold variation in auditory neurons responses recorded *in vivo* in barn owls. We found that spike threshold is quantitatively predicted by a model in which the threshold adapts, tracking the membrane potential at a short timescale. As a result, in these neurons, slow voltage fluctuations do not contribute to spiking because they are filtered by threshold adaptation. More importantly, these neurons can only respond to input spikes arriving together on a millisecond timescale. These results demonstrate that fast adaptation to the membrane potential captures spike threshold variability *in vivo*.

## Author Summary

Neurons spike when their membrane potential exceeds a threshold value, but this value has been shown to be variable in the same neuron recorded *in vivo*. This variability could reflect noise, or deterministic processes that make the threshold vary with the membrane potential. The second alternative would have important functional consequences. Here, we show that threshold variability is a genuine feature of neurons, which reflects adaptation to the membrane potential at a short timescale, with little contribution from noise. This demonstrates that a deterministic model can predict spikes based only on the membrane potential.

## Introduction

Neurons encode information in sequences of stereotypical action potentials or spikes. Spikes are all-or-none voltage deflections triggered when the membrane potential of a neuron crosses a threshold. *In vivo*, the spiking threshold, as measured as the voltage at the upstroke of spikes, varies with firing history and input properties. This phenomenon has been widely observed in the central nervous system, e.g. visual cortex [1], [2], auditory midbrain [3], hippocampus [4], somatosensory cortex [5]. It has been proposed that threshold variability measured in vivo reflects an adaptation of the spike threshold to the membrane potential, due to the inactivation of sodium channels [6]–[8] or the activation of potassium channels [9], [10]. Threshold adaptation would have a profound influence on how the combined input of a neuron is encoded in the spiking output [5], [6], [11]–[14], such as enhancing coincidence detection [1], [2], improving feature selectivity [5] and temporal coding in sensory neurons [15]. However, previous studies *in vivo* only reported correlations suggestive of threshold adaptation. Other authors have suggested that threshold variability observed *in vivo* could also reflect measurement artifacts because spikes are initiated at the axon initial segment but measured at the soma [16]. Threshold variability could also be due to channel noise [17], slow changes in excitability [18] or modulation by axonal synapses [19]. More generally the voltage measured at the upstroke of spikes may be a poor estimate of the actual criterion for spiking (which could depend on unobserved quantities). The goal of this study was to determine whether threshold variability observed *in vivo* is mainly due to threshold adaptation to the membrane potential, or to one of the alternative hypotheses.

Unfortunately, this question cannot be entirely addressed *in vitro*, where inputs are better controlled. First, there are potential sources of threshold variability *in vivo* that do not exist in vitro; in particular, noise and synaptic inputs to the initial segment. Second, properties of Na channels are likely to be different *in vivo*. Indeed, Na channels can be modulated in various ways, including their peak conductance and both the time constant and voltage-dependence of inactivation [20]. Therefore, results *in vitro* may not readily extend to *in vivo* conditions.

In this work we studied the dynamics of the spiking threshold in neurons of the barn owl's external nucleus of the inferior colliculus (ICx) *in vivo*. While the spatial tuning [21], [22] and the underlying computations in ICx neurons have been investigated [23]–[25], previous studies have shown wide variation in spiking threshold over the stimulus duration [3]. To understand this variability, we fitted a mechanistic model of spike threshold adaptation that generalizes a model based on sodium-channel inactivation [26] to intracellular recordings *in vivo*. The model is used to test whether it is possible to accurately predict spiking from the membrane potential history. If threshold variability is due to noise, then this prediction should fail; if it is due to factors other than adaptation (for example phosphorylation of Na channels, or GABA inputs onto the initial segment), then the parameter values of the fitted model should depend on the stimulus.

The model was able to predict spikes with high accuracy and to account for most observed variance in measured threshold. In addition, it allowed us to estimate the threshold at all times, including between spikes. We found that the spike threshold tracks the membrane potential at a shorter time scale than the membrane time constant. The “effective signal” for spike initiation is then best defined as the difference between threshold and membrane potential. Fast threshold adaptation has two major functional consequences: 1) the effective signal is less variable than the membrane potential, because low frequency components of the input are filtered out; 2) the neuron can only respond to inputs with dynamics faster than the adaptation timescale, an order of magnitude lower than the membrane time constant. These findings show that most threshold variability observed *in vivo* in these neurons can be explained by fast adaptation to the membrane potential.

## Results

### Spike threshold depends on preceding membrane potential

**Figure 1:**
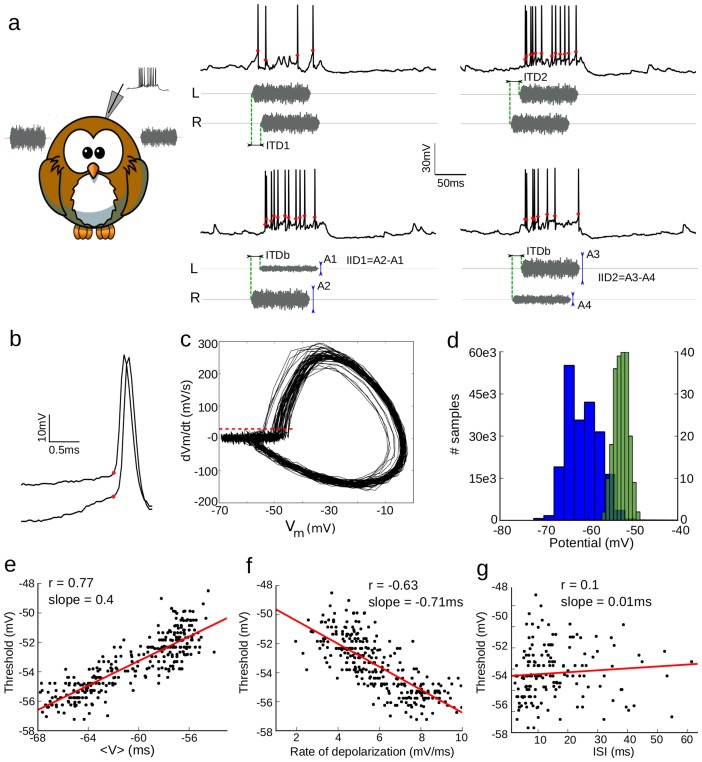
*In vivo* intracellular recordings. **a**, Intracellular recordings (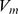) in the owl's ICx, with binaural stimuli (L: left, R: right). Either ITD is varied at best IID (top) or IID is varied at best ITD (bottom). Owl picture source: http://openclipart.org/detail/17566/cartoon-owl-by-lemmling. **b**, Two spikes from the traces in (a); red dots indicate the estimated spiking threshold. **c**, Trace from (a) shown in phase space: 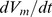 vs. 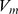. Spike threshold is detected when 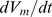 exceeds a fixed value (red dashed line). **d**, Distribution of subthreshold membrane potential (blue) and spike threshold (green). **e**, Spike threshold vs. average 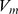 before spike. **f**, Spike threshold vs. depolarization slope before spike. **g**, Spike threshold vs. preceding interspike interval. Red lines are linear regressions.

Neurons of the barn owl's ICx are selective to sound direction [21], by combining tuning to interaural time (ITDs) and intensity differences (IIDs) [22], [23]. We recorded the membrane potential (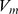) of ICx neurons *in vivo* while presenting 100 ms broadband sounds (white noises filtered between 0.5 and 10 kHz) through earphones, varying either ITD or IID (Fig. 1a). Spike thresholds (Fig. 1b) were measured using the 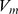 derivative, a procedure known to produce reliable estimates [27]. At spike initiation the derivative increases abruptly and the precise value of the criterion makes little difference to the estimated voltage (Fig. 1c). The spike threshold was highly variable, spanning a range of about 8 mV (σ = 3.1±1.1 mV). In fact, the distribution of spike thresholds was so large that it overlapped the 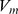 distribution induced by the input (Fig. 1d). As previously observed in this [3] and other areas [1], [5], [13], spike threshold was positively correlated (r = 0.75±0.1, regression slope = 0.43±0.1) with the average 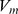 preceding spikes (Fig. 1e), and negatively correlated (r = 0.61±0.1, regression slope = −0.49±0.3 ms) with the rate of depolarization before spikes (Fig. 1f). We did not observe significant correlation between inter-spike interval (ISI) and spike threshold (r = 0.2±0.2, regression slope = −0.01±0.03 mV/ms, Fig. 1g), as was observed in a few other studies [4], indicating that spike refractoriness is shorter than typical ISIs.

### Fitting a spike threshold model

These observations suggest that the spike threshold adapts to the 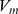 dynamics. However, what we called “spike threshold” above is in fact only a measurement of the voltage at the upstroke of spikes. It could be that the relevant criterion for spiking is a quantity (or set of quantities) other than somatic voltage, and that the voltage at the upstroke of spikes is correlated with 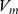 history but has no causal relationship therewith.

To address this issue and demonstrate that the spiking criterion (and not just the measured voltage at the upstroke of spikes) adapts to the membrane potential, we used a generalization of a model of threshold adaptation based on sodium channel inactivation [8] to predict the occurrence and timing of spikes. Our goal was to predict spike trains, not the voltage at the upstroke of spikes.

**Figure 2:**
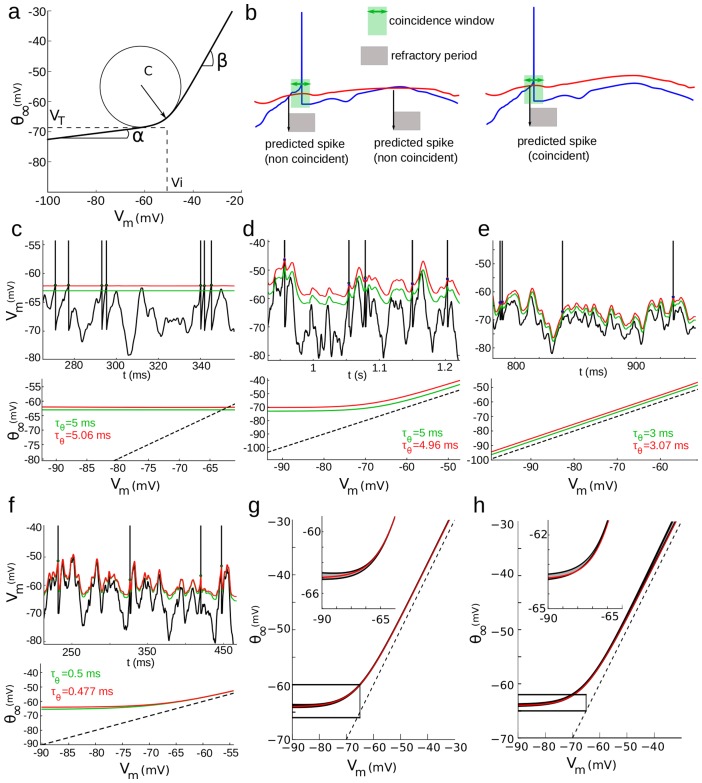
Model fitting approach. **a**, Steady-state threshold function, defined by 5 parameters. **b**, Illustration of the model fitness computation, Voltage trace (blue) and the corresponding dynamic threshold in the model (red). A spike is predicted when the curves cross, and a refractory period follows (grey). Prediction is considered correct when the actual and predicted spikes are within a fixed coincidence window (green). Left: incorrect predictions, right: correct prediction. Note that for the sake of illustration the coincidence window is drawn larger than what it is in reality. **c–f**, Top: output of the fitting procedure on neuron models with explicit dynamic threshold (green: actual dynamic threshold, red: model prediction), with four different steady-state threshold functions and threshold time constants (bottom). **g**, The fitting procedure was run for the same model shown in **f**, but with input currents varying in mean (20–200 pA) and standard deviation (50–400 pA). The shaded area shows the mean and standard deviation of the fitted steady-state threshold function: optimization results were not strongly dependent on the input current used for training. **h**, Same as **g**, but with 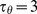 ms and input current with short autocorrelation time constant (0.5 ms).

Although the model was derived from properties of sodium channels, we used it here as a phenomenological model of threshold adaptation, which may also be consistent with other intrinsic mechanisms (see Discussion). This model consists of a differential equation describing the adaptation of the threshold 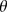 to a function of 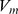, 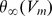, with a time constant 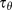:
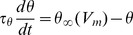
A spike is predicted to occur when 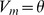. More generally, the spike threshold is defined as the voltage value at which the neuron would spike if its membrane potential were instantaneously brought above it. Thus it is a threshold in the sense of an explicit spiking criterion, unlike the empirical measurements. The function 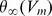, called the steady-state threshold, represents the value of the spike threshold when 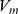 is clamped at a fixed value. This can be considered as a general first-order model of threshold adaptation. Theory based on the properties of sodium channels predicts that the steady-state threshold is constant below the half-inactivation voltage 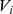, and increases approximately linearly above it [8]. However, threshold adaptation can also result from activation of voltage-gated potassium channels [9]. Therefore, to be more general, we did not impose a constant threshold below 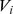. Instead, we used a smooth function with a different slope below and above the critical voltage 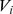, and a parameterized curvature (Fig. 2a). Parameters characterizing the two slopes, the connecting point and the curvature were constrained by the data. Some threshold adaptation models also include an explicit effect of spikes on threshold [26], [28] (the threshold increases after each spike), but it did not appear useful in our case, as we observed no correlation between ISI and spike threshold.

A straightforward approach would be to use this model to predict the value of spike threshold measured in the intracellular traces. However, as argued above, the measured somatic voltage at the upstroke of spikes may not correspond to the spike threshold, in the sense of a criterion for triggering a spike. For example, it has been argued that the relevant criterion should in fact be the voltage value at spike onset in the axon initial segment (AIS), where spikes are initiated [16], [29]. Even if the somatic voltage at the upstroke of spikes truly corresponded to the spike threshold, there would still be a methodological issue with optimizing the threshold model to predict that voltage. Indeed a trivial solution to the fitting problem is the threshold model defined by 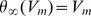 and 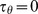 ms: the “spike threshold” always equals the membrane potential, in particular at the upstroke of spikes.

To avoid these problems, we instead used the threshold model to predict the occurrence of spikes and their precise timing based only on 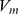. The trivial solution mentioned above is a poor predictor of spikes since it would predict too many spikes. The voltage trace was thus passed through the model equation to produce a dynamic spike threshold (Fig. 2b). Theory predicts that a spike should be produced when the voltage trace crosses threshold. The model can fail by producing spikes at the wrong time or by producing extra spikes. To account for both types of errors, we defined a stringent coincidence window (δ = 84 µs) and calculated the proportion of coincident spikes in both the recorded and predicted spike trains. We used the gamma factor 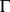, a normalized coincidence measure that has been used in a number of studies [30]–[32]. We optimized the model parameters to maximize 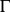 on a given recording, and the model performance was then tested on different recordings in the same cell.

We first checked that this optimization strategy was correct on different neuron models with an explicit adaptive threshold with a time constant of 3–5 ms (Fig. 2c–e; see Materials and Methods). The first model had a fixed threshold (Fig. 2c), the second an adaptive threshold with rectified-linear characteristics (only adapts above 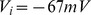; Fig. 2d) and the third a threshold that adapted linearly in the entire voltage range (Fig. 2e). Note that there is a constant bias in the predicted threshold, corresponding to the sharpness of spike initiation in these neuron models (i.e., spikes start slightly above the threshold value because sodium channels open gradually) [8]. Apart from this bias, both the steady-state threshold curve and the adaptation time constant were correctly estimated by the optimization procedure (Fig. 2c–e, bottom curves). We also confirmed that the fitting procedure yielded expected results when the threshold time constant was an order of magnitude shorter than the membrane time constant (Fig. 2f). Finally, we checked that the resulting parameters did not depend on the input statistics, by running the optimization procedure with input currents of different means and standard deviations on models with a short threshold time constant (Fig. 2g) and a short input autocorrelation time constant (Fig. 2h).

**Figure 3:**
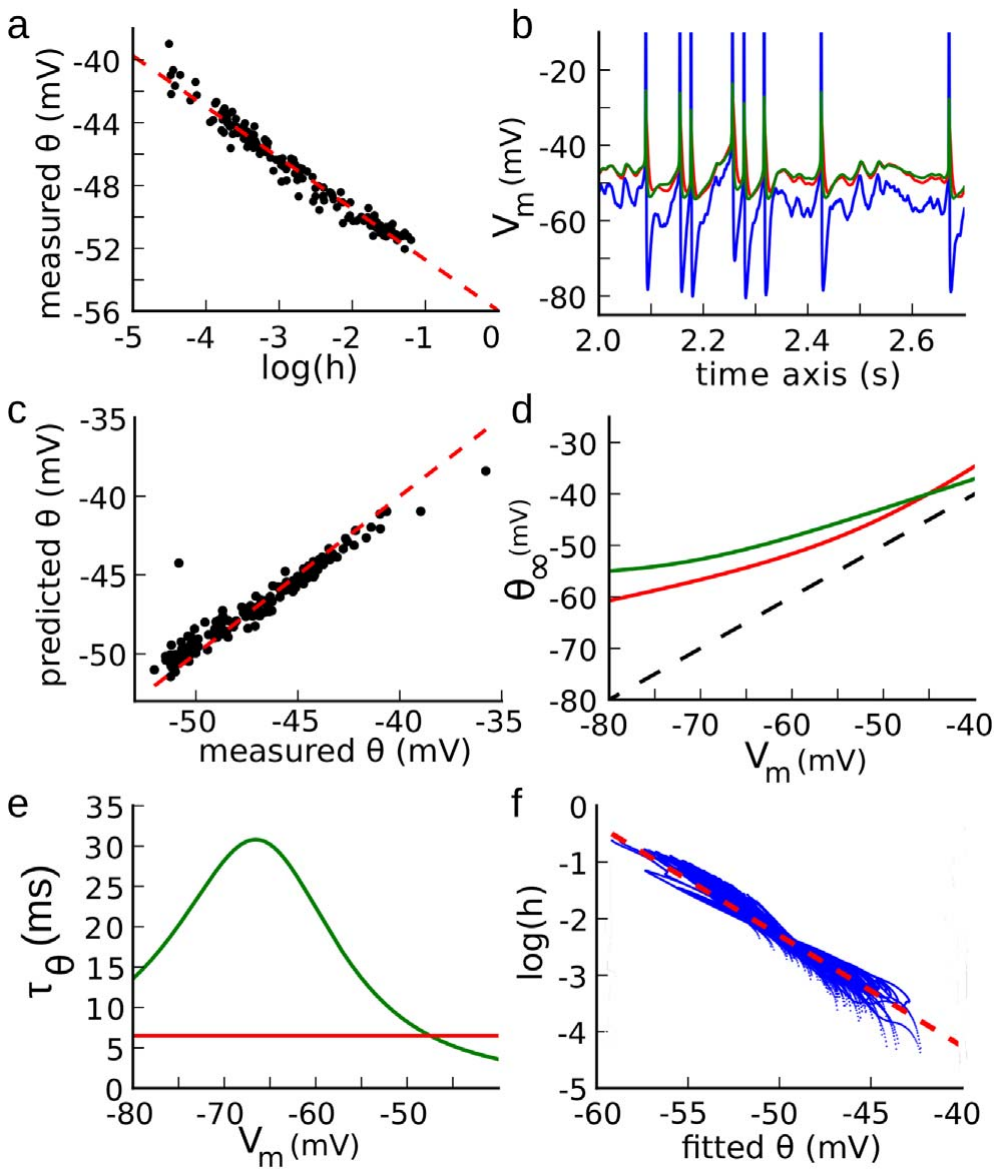
Fitting procedure applied on a multicompartmental model of a cortical neuron [**7**] **.** **a**, Spike threshold measured at the soma vs. logarithm of the sodium inactivation variable h at the axonal initiation site. The dashed line shows the linear regression (slope 3.2 mV). **b**, The fitting procedure is run on the somatic voltage trace (blue), and the predicted threshold (red) is compared to the threshold calculated from the value of ionic channel variables (green; as in [26]). **c**, Predicted threshold resulting from the fitting procedure vs. measured threshold for all spikes. The dashed line is the identity. **d**, Steady-state threshold function of the optimized model (red) compared to the corresponding function calculated from the properties of sodium channel inactivation. **e**, Estimated time constant of threshold adaptation (red) vs. time constant of sodium inactivation. The estimation is correct in the spike initiation zone (−50 to −40 mV). **f**, Logarithm of the sodium inactivation variable h at the axonal initiation site plotted against predicted threshold for the entire simulation, excluding spikes.

We then applied the fitting procedure to a biophysically detailed neuron model, in which spikes are initiated in the AIS and Na channel densities in the axon were measured with immunochemistry [7]. In this multicompartmental model, the value of the spike threshold measured at the soma can be accurately predicted from the value of ionic channel gating variables at the AIS [26]. We stimulated the model with fluctuating current, and we observed that there was a linear dependence between the measured value of the spike threshold and the logarithm of the Na inactivation variable h at the AIS (Fig. 3a; slope −3.2 mV; r = −0.98). We then fitted the threshold model to the voltage response of the model (Fig. 3b). After optimization, we observed that the time-varying threshold of the fitted model closely tracked the spike threshold estimated from ionic channel gating variables (which were hidden to the fitting procedure). At the spike times predicted by the fitted model, the corresponding predicted threshold was very close to the actual measured threshold (Fig. 3c). The steady-state threshold curve matched the curve calculated from the Na inactivation function [8], especially near the spike initiation region (Fig. 3d). In the multicompartmental model, the time constant of Na inactivation is voltage-dependent, unlike in our simple threshold model (Fig. 3e, green). However, the fitted threshold time constant matched the value of the inactivation time constant in the spike initiation region (−50 to −40 mV; Fig. 3e, red). Finally, we found that the value of the Na inactivation variable h at the AIS could be estimated between spikes from the value of the spike threshold in the fitted model (Fig. 3f; slope −0.22 mV^−1^; r = −0.97). Those results show that our method can successfully predict the spike threshold and characterize the sodium inactivation properties at the AIS of a complex multicompartmental neuron model containing an axon and an extended dendritic tree.

**Figure 4:**
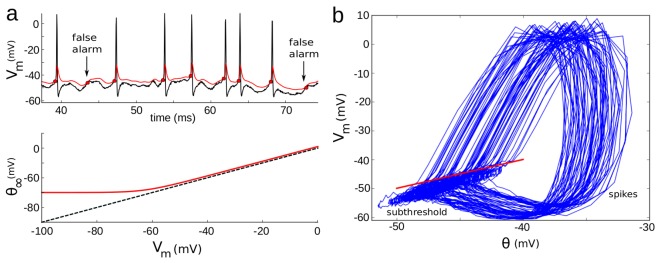
Fitting procedure applied on an intracellular voltage trace. **a**, Top: voltage trace (top, black) and predicted threshold (red). Bottom: steady-state threshold in the fitted model. **b**, 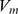 vs. predicted threshold for the trace in (a). The identity line (red) sharply separates subthreshold fluctuations from spikes.

Taken together, these results show that our optimization strategy can indeed accurately characterize the properties of spike threshold adaptation. We then applied this technique on our recordings, where spike times were accurately predicted, with few false alarms and typical rectified-linear curves for the estimated steady-state threshold (Fig. 4a). To emphasize the fact that we predict the threshold for spike initiation, and not simply the voltage at the upstroke of spikes, we show the voltage trace vs. dynamic threshold 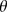 in Fig. 4b, where it can be seen that a spike is produced as soon as the identity line is crossed. Also, there are no crossings of the identity line between spikes. The absence of threshold crossings between spikes can be related to the sharpness of spike initiation [33], due to the compartmentalization of spike initiation in the AIS [34]. This observation means that the value of 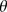 in the model predicts whether a spike is initiated, rather than simply predicting the somatic voltage at spike onset. This implies that it is indeed possible to predict spikes using only the membrane potential at the soma, even though spikes are initiated in the axon initial segment.

### Fitted parameters are consistent among conditions

**Figure 5:**
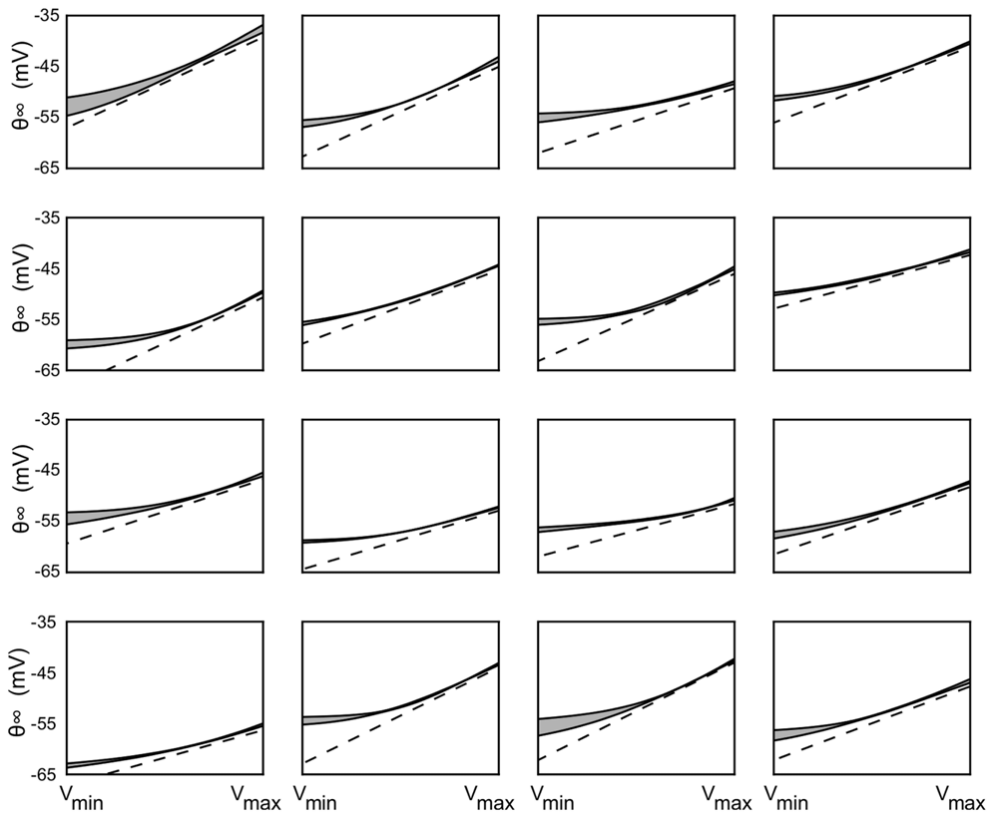
Steady-state threshold curves. Threshold curves resulting from optimizing the threshold model to recordings in 16 cells. The dashed line is the diagonal 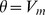 and the shaded area represents the average ± standard deviation over all recording conditions in each cell.

**Figure 6:**
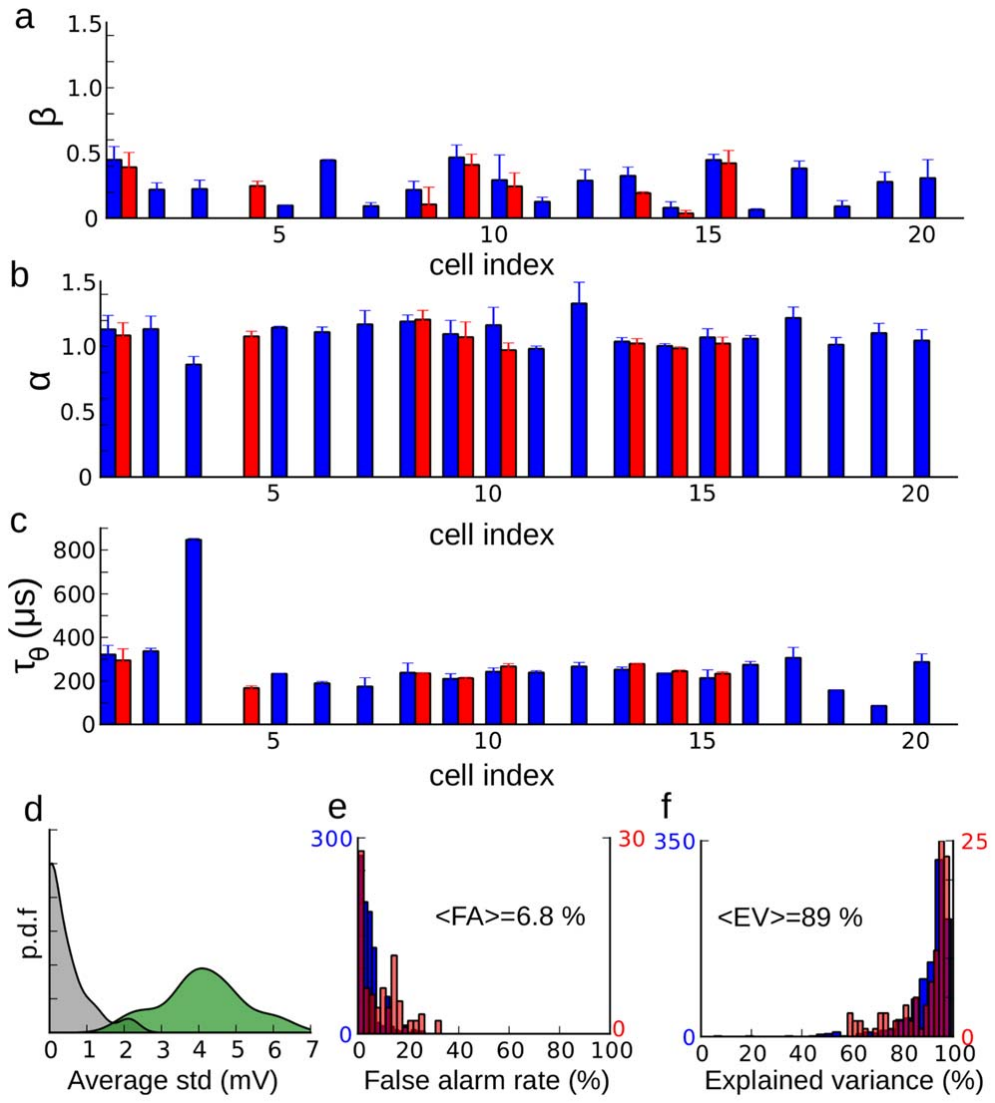
Fitting results. The optimization results for all cells are shown for three parameters: high voltage slope 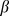 (**a**), low voltage slope 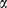 (**b**) and time constant 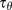 (**c**). Blue bars correspond to mean ± standard deviation over all recordings categorized by average membrane potential, and red bars (when available) correspond to mean ± standard deviation over all recordings categorized by stimulus condition (e.g. varying ITD with fixed IID). **d**, Distribution of average distance within cells between steady-state threshold functions (grey) and between steady-state threshold functions and the diagonal (green). **e**, Distribution of false alarm rates when the models are tested against recordings with a different mean 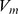 (blue) and with different sound stimulation (red) than used for fitting. **f**, Same as (d) for the explained variance of measured spike threshold.

If threshold variability is due to ionic channel properties, then threshold parameters should depend on the cell and not on the experimental condition. On the contrary, if threshold variability were due to other factors such as synaptic input onto the axonal initial segment, we would expect these parameters values to be variable across conditions. Therefore we optimized the model parameters separately on each cell and sound-stimulation condition (e.g., one condition is varying the ITD with a fixed IID) to check for stimulus dependency. As an additional check of robustness, we divided the entire set of recordings into subsets (2–8) with different 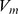 ranges, and optimized the model parameters separately in each set. We then compared the parameter values obtained for the same cell but different recordings. We found little variation in the results across conditions in the same cell (Fig. 5 and Figs. 6a, b, c). Consistent with theoretical predictions for Na channel inactivation [8], the steady-state threshold was near-constant at low voltages and increased linearly with slope near 1 at high voltages (Fig. 5). The fact that the steady-threshold curve does not cross the diagonal (Fig. 5, dashed lines) is consistent with large threshold variability [8].

**Figure 7:**
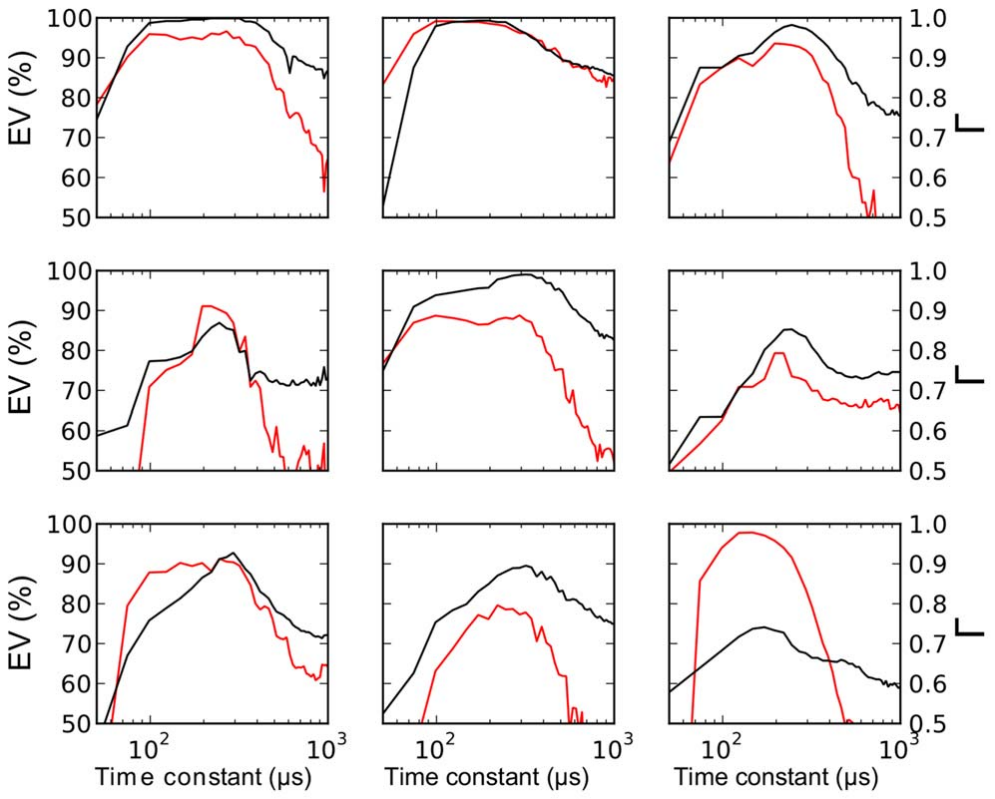
Fit quality vs. threshold time constant. To show that the optimized threshold time constant (about 260 µs on average) is accurate, we fitted the threshold model to the recordings while setting the time constant to a fixed value, i.e., the time constant is no longer a parameter to be optimized. The plots show the resulting gamma factor (in black, right ordinate) and explained variance (in red, left ordinate) as a function of threshold time constant for 9 cells. Moving the time constant away from its optimal value results in large increases in the fitting error.

In the entire set of cells (n = 21), we consistently found that the slope of the steady-state threshold curve was small at voltage smaller than 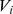 (Figs. 6a, mean 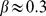) and near 1 above 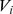 (Figs. 6b, mean 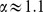), which is consistent with predictions based on sodium channel inactivation [8]. The mean critical voltage, 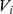, was −59±6 mV and the minimum threshold was 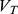 = −61±6 mV. Although there is some uncertainty about absolute voltage in intracellular recordings with sharp electrodes, the 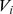 values are within the range of half-inactivation voltages of Na channels [35]. The curvature of the steady-state threshold is determined by the model parameter k_a_ = 7±2 mV, which is in the range of measured Na activation slopes [35]. Finally, the threshold-adaptation time constant was 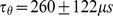 (Fig. 6c). Although this may seem small, time constants tend to be short in the barn owl's auditory brainstem, which is specialized for fast temporal processing [6], [13], as also seen in the timescale of spikes in Fig. 1 (see Discussion). In addition to the fact that threshold-adaptation time constants were similar across cells and recording conditions, the precise value of the model time constant was also important for predicting spikes. Fixing the time constant to a shorter or larger value than the optimal one significantly degraded the fitting quality (Fig. 7). A consistent observation is that above 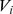, the steady-state threshold always lies just a few mV above 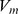 (Fig. 5, distance between solid curve and dashed line). Thus the condition for triggering spikes is not 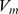 exceeding a fixed threshold, but rather a fast depolarization of a few mV. This property implies that when the neuron is slowly depolarized, it does not spike because the threshold increases at the same time. It can contribute in making the neuron respond with a single spike at the onset of a current step – but not necessarily because the reset may introduce fast variations in membrane potential. Electrophysiological properties of IC neurons are not known in the barn owl, but onset electrophysiological behavior has been observed in IC neurons of rodents, although not all neurons [36]. In the chick, neurons in Nucleus Laminaris, which project to IC, also respond to current steps by firing a single spike [37].

The optimized parameters varied across cells but not across stimulation protocols or 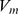 ranges in the same cell (Fig. 6a–c, blue error bars). The average distance between steady-state threshold curves obtained in the same cell for different conditions was an order of magnitude smaller than the average distance between steady-state threshold curves and the diagonal (Fig. 6d). These findings indicate that there is little threshold adaptation acting on a slow timescale in these neurons.

We then tested the optimized threshold models on recordings in the same cell that were not used for fitting the parameters, whether a different stimulation protocol or a different 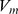 range, and we found that the models produced few false alarms (6.8%, Fig. 6e). Finally we tested whether at spike times, the value of the spike threshold variable in the model corresponded to the measured somatic voltage at the upstroke of spikes. We found that the model threshold could account for 89% of experimentally measured “spike threshold” variance on average (Fig. 6f). This means that the measured somatic voltage at spike onset does in fact correspond to the spike threshold, in the sense of a criterion for triggering a spike. In addition, since this value can be accurately predicted by our model, this result implies that the measured spike threshold is in fact determined by the 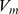 dynamics at the soma, rather than noise or external factors. Finally, it also implies that if there was stimulus-specific adaptation in these neurons as found in rats [38], it did not act on spike threshold, since we did not include such phenomena in the model.

### Functional consequences

**Figure 8:**
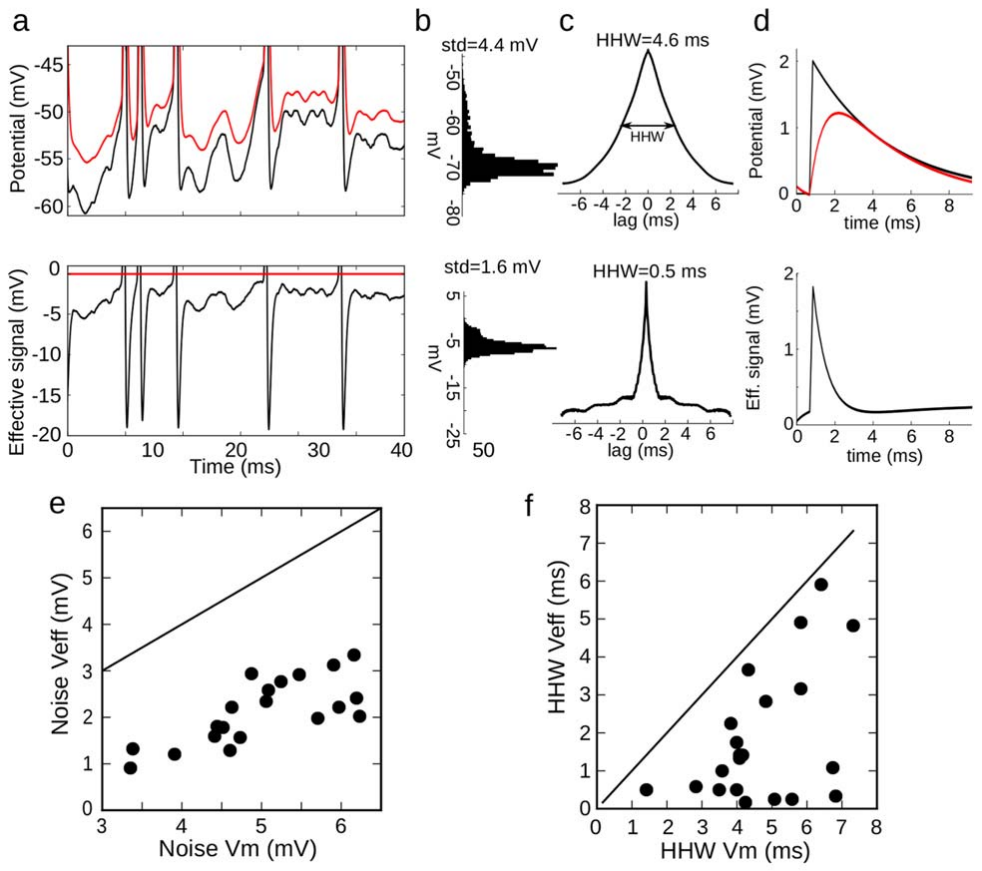
Effective signal. **a**, Top: voltage trace 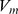 (black) and the corresponding fitted threshold (red). Bottom: the effective signal (black) is the difference. A spike occurs when it crosses 0 mV (red). **b**, Distribution of 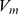 (top) and of the effective signal (bottom). **c**, Autocorrelogram of 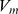 (top) and of the effective signal (bottom), showing the half-height width (HHW). **d**, Top: postsynaptic potential (PSP, black) and its effect on the threshold (red). Bottom: effective PSP. **e**, Standard deviation of the effective signal vs. standard deviation of 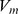 (line: identity). **f**, HHW of the effective signal's autocorrelogram vs. HHW of 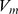
's autocorrelogram.

We finally turn to the functional implications of spike-threshold adaptation. Since the spike threshold adapts to 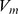, any voltage fluctuations that are slower than threshold adaptation should not have an impact on output spiking. This is captured by the concept of ‘effective signal’ (ES) illustrated in Fig. 8. The ES is the difference between the 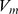 and the dynamic spike threshold (Fig. 8a). A spike is produced when the ES exceeds a fixed threshold (0 mV). Therefore, the 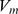 dynamics with threshold adaptation is equivalent to the ES dynamics with a fixed threshold. In the ES, voltage variability is greatly reduced, dropping from σ = 4.4 mV in the 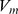 to σ = 1.6 mV in the ES for this recording (Fig. 8b). This occurs because slow voltage fluctuations are filtered out by threshold adaptation. This becomes clear when we compute the autocorrelation of the voltage traces (Fig. 8c). We found that the half-height width (HHW) of the 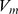 autocorrelation was 4.6 ms. This value corresponds to a membrane time constant of 3.3 ms for white noise input (HHW/(2.log 2)); in this case it may also reflect the timescale of synaptic currents. In contrast, the HHW of the autocorrelation of the ES is only 0.5 ms, which is in the order of magnitude of the threshold time constant. Because of threshold adaptation, postsynaptic potentials (PSPs) are effectively shortened. Specifically, the exponential decay of PSPs disappears from the ES, making the effective PSP shorter (Fig. 8d). In all cells, voltage variability is greatly reduced by threshold adaptation: from about σ = 5.1±1.0 mV in the 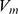 to σ = 2.2±0.8 mV in the ES (Fig. 8e). Since threshold adaptation has little effect on the peak size of a fast PSP (Fig. 8d), the ratio between PSP size and background voltage variability is effectively increased. In the same way, HHW is reduced from 4.7±1.4 ms to 1.7±1.5 ms (Fig. 8f). This means that the integration time window of these neurons is about three times shorter than inferred from the membrane potential alone, making the neuron sensitive to input coincidences at a millisecond timescale.

## Discussion

### Origin of threshold variability

*In vivo*, the spiking threshold is highly variable, typically spanning a range of about 10 mV. This phenomenon has been observed in many areas of the nervous system: visual cortex [1], [2], auditory midbrain [3], hippocampus [4], somatosensory cortex [5], neocortex [10], and prefrontal cortex [7]. Spike threshold has been found positively correlated with average membrane potential [2], [7] and inversely correlated with the preceding rate of depolarization [1], [2], [5], [12]. These observations are consistent with the hypothesis that the spike threshold adapts to the membrane potential, because of inactivation of sodium channels [1], [2], [5], [8], [26] and/or activation of low-voltage activated potassium channels (Kv1) [9], [10], [26]. However, these observations could also result in part or entirely from one or several of the following alternative causes:

- spike threshold variability resulting from ion channel stochasticity [17], or other independent sources of noise;
- experimental artifact where threshold appears variable at the soma but it is not at the spike-initiation zone in the axon [16], [39];
- spike threshold modulation by processes not directly dependent on 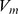, such as synaptic inputs to the axon initial segment (AIS) [19], intrinsic plasticity [18] or variations in total synaptic conductance [26].

Empirical support for threshold adaptation and for these alternative hypotheses comes from *in vitro* studies, and therefore it is not known whether and to what extent they may explain *in vivo* observations. Indeed, there are potential sources of threshold variability *in vivo* that do not exist in vitro (noise, synaptic inputs to the initial segment), and Na channels can be modulated in various ways, including their peak conductance and both the time constant and voltage-dependence of inactivation [20].

To distinguish between these hypotheses, we applied a predictive approach to *in vivo* recordings, which does not rely on measuring the somatic voltage at spike onset. Instead, the threshold model is evaluated on the basis of its ability to predict the occurrence of spikes from the previous membrane potential. This approach addresses the concern that criteria based on spike shape at the soma to measure “threshold” might inaccurately assess the actual criterion for triggering a spike.

In these data, the threshold model accounted for 89% of measured spike threshold variance. Therefore, most observed variability was due to deterministic processes, which ruled out hypothesis (a). It confirms theoretical considerations showing that ion channel stochasticity should imply a positive correlation between rate of depolarization and spike threshold, contrary to our and previous experimental observations [8].

According to hypothesis (b), spikes are actually initiated at a fixed voltage threshold, but it appears variable because it is not measured at the initiation site (in the axon). Our results discard this possibility because the threshold model is optimized to predict the occurrence of spikes, not the measured voltage at spike onset at the soma. It indeed predicts the occurrence and precise timing of spikes very accurately, and with very few false alarms. Therefore, the variability of measured somatic voltage at spike onset did reflect the variability of spike threshold in these recordings (see also Fig. 4b). It confirms theoretical considerations showing that variability due to hypothesis (b) should also imply a positive correlation between rate of depolarization and spike threshold [8].

To address hypothesis (c), we fitted the threshold model in the same cell but in different experimental conditions (either different ranges of 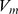 or different stimulus conditions). If threshold variability were due to other processes that are not directly determined by 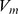 (e.g. synaptic input to the AIS or intrinsic plasticity), then we would expect the fitting process to yield different parameters values depending on context. In contrast, parameter values of the model were very robust across different conditions for the same cell, and variable between cells. These results make hypothesis (c) implausible in our recordings. On the basis of single-compartment biophysical models, it has been proposed that the total synaptic conductance may also modulate the spike threshold in a logarithmic way, by opposing the Na current [26]. Our results would only be consistent with this hypothesis if total synaptic conductance were constant in all conditions (all stimuli and all mean 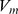). Although it seems unlikely, we cannot entirely rule out this possibility. Recent theoretical analysis taking into account the axonal initiation of spikes indicates that the total synaptic conductance at the soma should have negligible impact on spike threshold because spike initiation is compartmentalized [34] (i.e., only channels expressed at the AIS can directly modulate the spike threshold).

Therefore, our results discard all the alternative hypotheses mentioned above, and demonstrate that threshold variability reflects deterministic adaptation of the spike threshold to the somatic membrane potential.

### Biophysical mechanisms

Adaptation of spike threshold points to voltage-gated ion channels expressed in the AIS. Spike initiation is due to Na channels of the Nav1.6 subtype expressed in the distal part of the AIS [40]. These channels are partially inactivated at rest, and therefore voltage changes should substantially modulate the spike threshold by changing the proportion of available channels for spike initiation. The threshold model used in this study derives from a theoretical analysis of the biophysical properties of Na channels [8], [26]. This analysis accurately predicted the spike threshold in a multicompartmental model of a cortical neuron with measured channel densities in the AIS [26]. The theory predicts that 1) the spike threshold is constant in the hyperpolarized range because Na channels are not inactivated, 2) the spike threshold follows the membrane potential in the depolarized range because activation and inactivation curves have similar slopes [8], 3) the transition between the two regimes occurs at around half-inactivation voltage. Our results confirm these predictions.

The time constant of threshold adaptation may seem surprisingly low, about 250 µs. In Hodgkin-Huxley models, this adaptation time constant reflects the time constant of the underlying ionic channel mechanism (inactivation of Na channels or activation of K channels). Na channel inactivation time constants for subthreshold voltages are generally found to be on the order of the ms *in vitro*, in the cortex and hippocampus [41]. However, there is evidence that the time constant of inactivation can be modulated [20], and that it depends on functional constraints, such as energetic efficiency [41]. In the electric organ of the electric fish, it has been found the inactivation time constants of Na and K channels are co-regulated, and correlate with the frequency of electrical discharges [42]. In this particular context, Na inactivation time constant varied between 500 µs and 3 ms (Fig. 7). Therefore it seems possible that this time constant is also short in a nucleus involved in the processing of sounds with frequencies of several kHz. The fact that spikes are shorter than 500 µs (Fig. 1b) in our recordings is an indication that it may indeed be the case.

Low-voltage activated potassium channels (Kv1) are also expressed at high density in the AIS [43], [44]. Activation of Kv1 channels by depolarization can also raise the threshold, and therefore, Kv1 channels can produce threshold adaptation with similar qualitative properties as Na channel inactivation [26]. A few *in vitro* studies show that pharmacologically blocking Kv1 channels can abolish threshold variability [9]. This could be because Kv1 channels are responsible for threshold adaptation, or because blocking these channels lowers the spike threshold so that spikes are initiated before Na channels can inactivate (this happens in Fig. 3 if threshold curves are shifted down and intersect the diagonal). It is possible that the residual threshold adaptation seen in the hyperpolarized range (Fig. 5) is due to Kv1 channels. Clearly distinguishing between Na inactivation and Kv1 activation might require dual recordings in the soma and AIS, sodium imaging or pharmacological manipulations.

### Threshold variability in other areas

Our results were obtained with *in vivo* intracellular recordings in the barn owl's inferior colliculus, and one may wonder to what extent they may generalize to other areas. The detailed statistics of threshold variability are similar to previous observations in cortical neurons [1], [5], both qualitatively and quantitatively, except perhaps for the depolarization rates, which tend to be larger in our recordings (Fig. 1f). The mechanisms of spike initiation are also widely shared across the nervous system [40], [44]. Therefore it is reasonable to expect that our findings are generally valid. However, it is likely that the time constant of threshold adaptation (which was only a few hundred of microseconds in our study) is larger in other areas. Indeed auditory neurons in subcortical areas are known to display faster kinetics than in other areas, not only in the barn owl but also in mammals [45], [46].

Another likely difference is that in some *in vivo* studies, spike threshold was found to strongly depend on the time since the previous spike [4], [28]. This is not contradictory with the model, which displays this phenomenon when the adaptation time constant is larger than the typical interspike-interval. Finally, in pyramidal cells of the cortex, and also in hippocampus neurons, the AIS is targeted by GABAergic neurons named Chandelier cells [19]. Their action could potentially modulate the spike threshold depending on local network activity (for instance on the phase relative to theta oscillations in the hippocampus [47]), in a way that is not determined by the cell's Vm at the soma (hypothesis (c)).

### Signals that elicit spikes

Our results show that threshold variability is mainly due to deterministic features of the input, rather than noise. Given the extent of this variability (more than 10 mV), this finding has major implications for the input-output properties of neurons. It implies that the relevant time-dependent variable is not so much the membrane potential, but rather its distance to a dynamic threshold, which we called the “effective signal”.

Our method allowed us to estimate the spike threshold not only at spike times but also continuously between spikes, and thus to estimate the effective signal. We found that a large part of the variability appearing in the voltage trace vanishes in the effective signal, because slow variations of the membrane potential are filtered out by threshold adaptation, leaving only variations that are faster than threshold adaptation. Secondly, we found that the effective signal varies on a shorter timescale than the membrane potential. It implies that the temporal window of integration is shorter than expected from the membrane time constant, and closer to the threshold time constant. These findings confirm previous suggestions that threshold variability enhances coincidence detection properties of cortical neurons [1], [5], and corroborate observations that spikes tend to be preceded by fast depolarizations in cortical neurons *in vivo* [48].

Taken together, these findings demonstrate the causal link between membrane potential dynamics and spike threshold variability *in vivo*. In elucidating the deterministic nature of threshold, this work shows that threshold adaptation makes neurons selective to fast input variations and remarkably insensitive to slow ones.

## Materials and Methods

### Ethics statement

The protocol #20110502 for this study followed the National Institutes for Health Guide for the Care and Use of Laboratory Animals and was approved by the Institutional Animal Care and Use Committee of California Institute of Technology.

### Experimental methods

Data were obtained from *in vivo* intracellular recordings of 21 ICx neurons in 14 anesthetized adult barn owls, as described previously [24], [24,49]. Sharp glass electrodes (40–80 MΩ) filled with 2M potassium acetate were used for recording. All experiments were performed in a double-walled sound-attenuating chamber. Acoustic stimuli were digitally synthesized and delivered through earphones. Sound stimuli consisted of broadband-noise bursts (0.5 to 10 kHz, 100 ms in duration and 5 ms linear rise/fall times, 30 dB above threshold) presented once per second. Earphone assemblies containing a speaker and a calibrated microphone were inserted into the ears and gaps were sealed with silicone material. The earphones were calibrated at the beginning of each experiment to correct for speaker irregularities. Intracellular recordings were stored at 24 kHz sampling rate.

### Spike threshold measurement

Measured spike threshold is defined as the voltage at the onset of action potentials. For each spike, the onset is defined as the first time preceding the peak when the first derivative 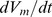 crosses a fixed criterion, 25 mV/ms. On the phase plot (Fig. 1c), it corresponds to a voltage value that is only crossed when a spike is produced. The precise value is not critical for model fitting because we predict the timing of spikes rather than the voltage at spike onset. Fig. 1e shows the mean 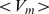 computed in 5-ms window preceding a spike. Fig. 1d shows the rate of depolarization over 1.5 ms preceding a spike.

### Adaptive threshold model

The dynamic threshold depends only on 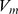, and is determined by [26]:
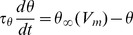
Where 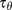 is the time constant of the threshold dynamics. 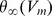 is the steady-state threshold (Fig. 2a):
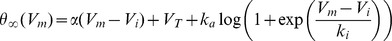
where 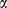 is the slope on the left side of the knee. The slope 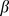 on the right side is 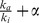. The curvature C (Fig. 2a) is indirectly determined by 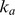, 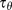, 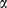, 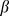, 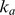, 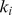, 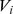, 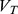 were the parameters to optimize. A spike is produced when 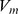 exceeds 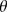 and is followed by a refractory period of 0.5 ms. If threshold modulation is due to sodium channel inactivation, the theoretical prediction [8] corresponds to 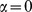.

### Model fitting procedure

Given a 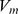 trace and its corresponding spike onsets (described above), we want to find the parameter values of the adaptive threshold model that maximize the similarity between predicted and recorded spike trains. This similarity is quantified using the gamma factor (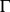) [30], [31], a normalized measure of coincidence between spike trains within a temporal window 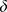:
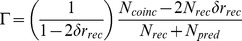


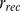
 is the mean firing rate of the experimental recording, 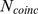 is the number of coincidences between the predicted and recorded spike trains computed within a time window 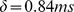, 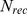 and 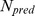 denote the number of spikes in the recorded and predicted spike train, respectively. 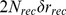 is the expected number of coincidences generated by a Poisson process with rate 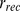. The first term in brackets is a normalization factor so that the maximum of 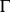 is 1. 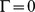 means that there are no more coincidences than expected by chance whereas 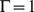 means that the model prediction is perfect, at temporal resolution 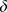.

To perform the optimization, an evolution algorithm (CMAES) [50] was implemented on Graphical Processing Units (GPU) [32], [51] using the Playdoh optimization toolbox [52].

### Neuron models

A spiking neuron model with an adaptive threshold was used to generate the membrane voltages of Fig. 2c–h. All other voltage traces are intracellular recordings. The model is based on the exponential integrate and fire [53]. 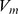 is governed by a differential equation that includes a leak current and an exponential term describing sodium current activation at spike initiation:
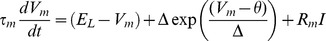
where 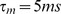 is the membrane time constant, 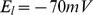 is the reversal potential of the leak current, 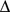 characterizes the sharpness of the initiation. 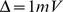 except in Fig. 2 f., g. and h. where 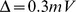. 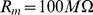 is the membrane resistance. The membrane voltage diverges quickly once it exceeds the threshold 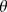, it is then reset to −70 mV, and a refractory period of 0.8 ms follows (in practice, spikes are detected when 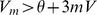). In Fig. 2c–f, the input current *I* is an Ornstein-Uhlenbeck process with mean 40 pA, standard deviation 120 pA, and time constant 3 ms. In Fig. 2g–h, the optimization is performed on a set of currents with mean between 20 and 200 pA and standard deviation between 50 and 400 pA, selecting those eliciting at least 20 spikes and a firing rate lower than 200 Hz. Current time constant was 3 ms in Fig. 2g and 0.5 ms in Fig. 2h. The exponential model accurately captures the dynamics of the sodium current near spike initiation [28], while allowing sharp spike initiation. We used this, rather than a Hodgkin-Huxley model, because spike initiation is unrealistically shallow in a single-compartment Hodgkin-Huxley model and spike onsets are not well defined [33], [34]. Multicompartmental models can display sharp spike initiation [7] but the threshold is not explicitly defined, a problem to test the predictive power of a threshold model.

We assume that threshold dynamics are governed by the differential equation given in section “Adaptive threshold model”. The model has a constant threshold in Fig. 2c (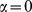, 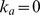, 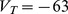 mV, 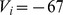 mV and 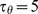 ms), rectified threshold in Fig. 2d (same except 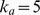 mV), linear threshold in Fig. 2e (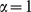, 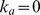, 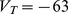 mV, 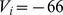 mV and 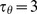 ms), rectified threshold in Fig. 2f, but with fast threshold adaptation (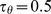 ms).

In Fig. 3, we used a biophysically detailed multicompartmental model of a cortical neuron based on immunochemistry measurements, in which spikes are initiated in the axonal initial segment [7]. It was stimulated at the soma with fluctuating current as described above, with mean 0.7 nA, standard deviation 0.2 nA and time constant 10 ms. The spike threshold is estimated from ionic channel gating variables as described in [26] (Fig. 3b, green).

All simulations except for the multicompartmental model were performed using the Brian simulator [54] with a sampling frequency of 42 kHz. The multicompartmental model was simulated with Neuron [55].

### Training and testing the threshold model

For each cell, the voltage traces were grouped in subsets. A subset is a set of traces sharing common conditions. The first type of condition used to characterize subsets is the binaural protocol used. For instance, the first subset can be the set of traces recorded when varying the ITD, another when varying IID, and another when varying average binaural intensity (ABI). Depending on the cell, there were two or three recording protocols used, resulting in two or three subsets. The second type of condition is the mean 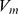 during stimulation. For each cell, responses to all sounds are ordered by mean 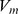. Each subset is then constructed incrementally by adding consecutive traces until there are at least 120 spikes in the subset. This makes 2–8 subsets per cell.

The prediction performance is quantified using two metrics. The false alarm rate (FA), reported as a percentage, is defined as the number estimated spikes that are not coincident with recorded spikes divided by the total number of recorded spikes. The explained variance (EV) quantifies the prediction quality of the voltage at spike onset:
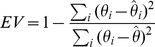
with
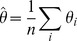
where 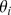 is the voltage at spike onset in the recorded trace and 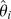 is the predicted voltage at spike onset. These two metrics were always used on recordings not used for fitting the model (different binaural protocol or different mean 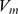).

For each cell, we calculate the average distance between steady-state functions 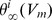 obtained for different conditions (Fig. 6d) using the following formula:
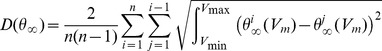
Where 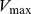 and 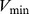 are respectively the maximum and minimum sub-threshold voltages in the trace under consideration, and n is the number of conditions. For comparison, we also report the average distance to the diagonal 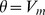: